# State Divorce Laws, Reproductive Care Policies, and Pregnancy-Associated Homicide Rates, 2018-2021

**DOI:** 10.1001/jamanetworkopen.2024.44199

**Published:** 2024-11-08

**Authors:** Kaitlin M. Boyle, Wendy Regoeczi, Chase B. Meyer

**Affiliations:** 1 Department of Criminology and Criminal Justice, University of South Carolina, Columbia; 2 Department of Political Science, University of South Carolina, Columbia

## Abstract

**Importance:**

Barriers to divorce and reproductive health care can threaten the health and safety of pregnant and recently pregnant females.

**Objective:**

To examine barriers to divorce and reproductive health care (access to contraception, family planning services, and abortion), and pregnancy-associated homicide rates in US states over a 4-year period (2018-2021).

**Design, Setting, and Participants:**

In this cross-sectional study, bivariate tests and regressions were used to analyze crude rates of pregnancy-associated homicide from the National Violent Death Reporting System in 181 state-years for calendar years 2018 to 2021, with analyses conducted on September 8, 2024, and updated in October 2025.

**Exposures:**

Barriers to divorce while pregnant and reproductive health care over a 4-year period in the US.

**Main Outcomes and Measures:**

Primary outcomes (pregnancy-associated homicide by intimate partners vs non–intimate partners and rates among younger Black, Hispanic, and White females) were assessed using the National Violent Death Reporting System. Negative binomial regression was used to test 2 hypotheses: barriers to divorce while pregnant and reproductive health care are associated with pregnancy-associated homicide rates.

**Results:**

Individual level data, including exact sample size, were not available in this study of state-level homicide rates. Negative binomial regression analysis showed that, where there are documented barriers to finalizing divorce during pregnancy, intimate partner homicide rates (incidence rate ratio [IRR], 2.11; 95% CI, 1.09-4.08; *P* = .03) and rates among younger (age 10-24 years) White females (IRR, 2.39; 95% CI, 1.12-5.09; *P* = .02) were significantly higher. In state-years with greater access to reproductive health care, rates were significantly lower for non–intimate partner homicide (IRR, 0.92; 95% CI, 0.87-0.98; *P* = .01) and for younger Black females (IRR, 0.91; 95% CI, 0.87-0.96; *P* < .001) and younger Hispanic females (IRR, 0.87; 95% CI, 0.79-0.96; *P* = .007).

**Conclusions and Relevance:**

In this cross-sectional study of pregnancy-associated homicide rates, barriers to divorce were associated with higher homicide rates and access to reproductive health care was associated with lower homicide rates. This study highlights the association between state legislation and pregnancy-associated homicide in the US, which is important information for policymakers.

## Key Points

**Question:**

Are barriers to divorce during pregnancy and reproductive health care access correlated with pregnancy-associated homicide rates in the US?

**Findings:**

In this cross-sectional study of state-level pregnancy-associated homicide rates from 2018 to 2021, rates were significantly higher in state-years with documented barriers to finalizing divorce during pregnancy and with more barriers to reproductive health care. The specific barriers associated with homicide risk varied based on how the person killed knew the suspect, and the racial and ethnic group of the person killed.

**Meaning:**

The findings of this study suggest that, in the US, barriers to divorce and reproductive health care pose a serious health risk to pregnant or recently pregnant females, given their association with homicide risk.

## Teaser

This cross-sectional study examines the association of barriers to divorce during pregnancy and access to reproductive health care with rates of pregnancy-associated homicide.

## Introduction

Homicide is a leading cause of death for pregnant women in the US.^1,2,3,4,5,6^ More women die from pregnancy-associated homicide (while pregnant or in the year after pregnancy), than other causes of maternal mortality, such as preeclampsia or hemorrhage.^7,8^ Rates of pregnancy-associated homicide vary by age and race, with individuals younger than 25 years and Black women at highest risk,^1,2,3,4,5,6,9,10,11^ and the largest proportion is committed by an intimate partner.^3,4,6^

While increased access to divorce has been shown to decrease rates of intimate partner homicide (IPH) in general,^12,13^ divorce barriers could also shape pregnancy-associated IPH if they pose obstacles to finalizing a divorce during pregnancy. Recently, barriers to finalizing divorce while pregnant in Missouri were highlighted, where anyone petitioning for divorce must disclose pregnancy, and a pregnancy may delay finalizing divorce. Since Missouri State Representative Ashley Aune called attention to this, journalists have highlighted additional states with similar barriers in place, where there is no legal prohibition to divorce while pregnant, yet judges are known to wait until after the child’s birth to finalize a divorce, a practice that could worsen intimate partner violence (IPV).^14,15,16^

Intimate partner violence and pregnancy-associated homicide are also intertwined with access to reproductive health care. Unintended pregnancies are more likely to occur in violent relationships,^17,18^ as they frequently involve a complex web of psychological, emotional, and/or sexual abuse.^19,20,21^ Reproductive coercion is one form of IPV that can result in unintended pregnancies through rape, sexual assault, sabotaging contraception, and restricting women’s contraception use through threats, violence, coercion, or control.^22,23,24^ Women who experience IPV during their pregnancy are more likely to miss prenatal appointments or delay starting prenatal care until late in their pregnancy,^18,25,26^ and there is a higher risk of pregnancy-associated homicide among women who receive no or late prenatal care.^5^ Barriers to reproductive care and delays in starting prenatal care have major implications. In many states, women lack options if they do not seek care until after passing a particular gestational stage. As of this writing in 2024, 17 states have passed total abortion bans or abortion bans at 6 weeks, before women typically have their first prenatal appointment.^27^

In 2022, the US Supreme Court overturned *Roe v Wade*, which provided federal protections for abortion access; when it was overturned, states were able to fully ban the procedure. Although there were not outright bans during the years 2018 to 2021, the period of current study, there were more than 350 state restrictions in place, preventing full access to abortion services even before *Roe v Wade* was overturned.^28^ Research conducted prior to the overturning of *Roe v Wade* found that individuals’ access to abortion is associated with a reduction in violence by the man involved with the pregnancy,^29^ and on the state level, states with more abortion barriers report higher rates of both maternal mortality^30^ and infant mortality.^31^ This association is not only isolated to nonviolent causes of death. In recent studies, rates of IPH were higher in states with more laws that regulate abortion providers,^32^ and states categorized as restrictive of abortion access had higher rates of peripartum homicide.^33^

To our knowledge, the current study is the first that examines the correlation between state-level barriers to divorce and pregnancy-associated homicide rates. The current study also expands previous research by examining the association between pregnancy-associated homicide rates and a range of both restrictive and expansive state-level policies on reproductive health care that vary year to year including, but not limited to, abortion. We examine these patterns over time (calendar years 2018-2021), including pregnancy-associated homicide rates for those killed by an intimate partner and those killed by someone other than an intimate partner. We also examine rates of pregnancy-associated homicide across racial and ethnic groups among younger females (age 10-24 years), as research reports significant differences in rates across racial and ethnic groups,^9,11^ and the highest rates are found among younger women.^8^ Based on previous research, this study tested 2 hypotheses. The first hypothesis is that states in which there are documented barriers to finalizing divorce while pregnant will have higher rates of pregnancy-associated homicide. The second hypothesis is that states with greater reproductive health care access (including contraception, family planning services, and abortion) will have lower rates of pregnancy-associated homicide.

## Methods

The Centers for Disease Control and Prevention administers the National Violent Death Reporting System (NVDRS).^34^ The NVDRS integrates data on violence from multiple sources, including law enforcement, medical examiners/coroners, and death certificates, and includes data on homicide, suicide, firearm-related deaths, deaths from legal intervention, and other causes of violent death. The dataset includes more than 600 data elements and thus provides considerable detail about individual incidents, including information about the injury, circumstances, and extensive demographic information. Beginning with 40 states at the start of the study period (2018), the NVDRS contained 3 additional states in 2019 and included data from 49 states in 2020 and 2021 (Washington, DC, and all states except Florida and Hawaii). Because data were collected in each participating state and year, the unit of analysis is the state-year (N = 181 state-years). Given its use of secondary, aggregated data, the study received exempt status from the institutional review board of the University of South Carolina. This study followed the Strengthening the Reporting of Observational Studies in Epidemiology (STROBE) reporting guideline.

### Pregnancy-Associated Homicide Rates

The Web-Based Injury Statistics Query and Reporting System (WISQARS) calculates crude rates of violent deaths as captured in the NVDRS. Various filters can be selected in WISQARS, including whether the individual was pregnant at the time of death or within a year of their death. WISQARS was used to calculate the crude rate of intimate partner and non–intimate partner pregnancy-associated homicide rates in each state-year for females from all age groups, although WISQARS calculates this rate per 100 000 females of reproductive age (15-49 years). WISQARS was also used to calculate crude rates for females aged 10 to 24 years who are at higher risk for pregnancy-associated homicide, across racial and ethnic identity: non-Hispanic younger Black females, younger Hispanic females of all races, and non-Hispanic younger White females. In the NVDRS, the females’ race and ethnicity is coded based on how it was reported in the source documents provided (eg, death certificate or medical examiner’s report).

### Barriers to Finalizing Divorce

A viral Instagram post in February 2024 claimed that pregnant people cannot file for divorce in Arkansas, Arizona, Missouri, and Texas, and news publications highlighted barriers to divorce during pregnancy, for instance, so that paternity can be confirmed and child support can be arranged before the case is finalized.^14,15^ In February 2024, *USA Today* conducted a fact check in which legal experts in these states were consulted, and the viral Instagram post was labeled “true.”^16^ In March 2024, they updated their rating as “partly false” and indicated Arizona should not be considered in this group.

*USA Today* further updated their post in February 2025, noting that while “divorces cannot be finalized in Texas while a woman is pregnant, as noted in legal paperwork widely used in state divorce proceedings,” the other states do not have “legal prohibitions” on divorce while pregnant, but that “their judges overwhelmingly—if not entirely—hold off on finalizing divorces until pregnancies are done for technical or procedural reasons.” Based on the currently available material, we coded Arkansas, Missouri, and Texas as having documented “barriers to finalizing divorce” (n=8 state-years, 4%).

### Reproductive Health Care Access

A recent study^35^ published state-level policies that restricted and expanded access to reproductive health care from 2006 to 2021. The authors analyzed 20 expansive policies, for instance, related to insurance coverage, Medicaid, protections for minors and dependents, and who can dispense contraception, along with 3 restrictive policies, such as restrictions on state family planning funds. For each state-year, count variables were calculated for expansive policies and for restrictive policies.

The Guttmacher Institute produced a series of annual reports that identify states in which abortion barriers are in place, which included license requirements (eg, who can perform an abortion), gestational limits, partial-birth abortion restrictions, restrictions on public funds and private insurance, mandatory counseling, mandatory waiting periods, and laws that permit individual health clinicians or institutions to refuse care; and requirements that parents consent to a minor’s abortion or are notified about a minor’s abortion.^27^ For each state-year, a count variable was calculated, summing the number of types of abortion barriers, ranging from 0 to 9.

Because measures of reproductive health policies and abortion barriers are highly correlated (ρ = −0.74; *P* < .001), an index was created for regression analysis. For each state-year, the number of restrictive reproductive health care policies and abortion barriers were subtracted from the number of expansive reproductive health care policies. This results in a Reproductive Health Care Access Index ranging from −8 to 17 (mean [SD], 3.227 [6.630]), with higher scores indicating greater access. For example, if a state-year had every abortion barrier (9) and every restrictive health care policy (3) and 0 expansive policies (0 of 20), their Reproductive Health Care Access Index score would be −12. A state with no abortion barriers or restrictive health care policies and every expansive policy would have a score of 20.

### Statistical Analysis

Data analysis was conducted on September 8, 2024. Like other violent crimes, pregnancy-associated homicide rates are not normally distributed, which was confirmed by Shapiro-Wilk tests of normality. The *z* scores ranged from 7.12 to 9.67, and all reached significance, indicating nonnormality (*P* < .001). Due to nonnormality, for bivariate analysis, the Wilcoxon rank-sum test was used to determine whether homicide rates were significantly higher in states where barriers to finalizing divorce during pregnancy have been documented. This is a common nonparametric test used for independent samples and dichotomous independent variables. The *z* statistic and significance levels are reported. To determine whether homicide rates are correlated with the number of restrictive and expansive reproductive health care policies and abortion barriers, variables that range from 0 to 20, Spearman correlation was used, which is an appropriate test to detect associations between continuous variables with nonnormal distributions.

Negative binomial regression was used because the dependent variables are nonnormal and overdispersed. A control variable for location in the southern US and fixed effects for years are included, given regional and year-to-year variation in reproductive health care access. Robust SEs are clustered by state in these 2-sided unpaired tests. Analyses were completed using Stata Software, release 17 (StataCorp LLC).

## Results

Individual level data, including exact sample size, were not available in this study of state-level homicide rates. Pregnancy-associated homicide rates, calculated per 100 000 reproductive-aged females in each state-year, are listed in Table 1. Mean (SD) IPH (0.11 [0.18]) was higher than non-IPH (0.03 [0.08]). For racial and ethnic groups, the highest rates were found among younger Black females (0.43 [1.02]), followed by younger Hispanic females (0.10 [0.41]) and younger White females (0.03 [0.07]).

**Table 1.** Barriers to Finalizing Divorce While Pregnant and Pregnancy-Associated Homicide Rates per 100 000 Females of Reproductive Age

Variable	Mean (SD) [range]	
	All state-years (N = 181)	Documented barriers to finalizing divorce (n = 8 state-years)
Intimate partner rate	0.11 (0.18) [0.00-1.62]	0.26 (0.14) [0.04-0.51]
Non–intimate partner rate	0.03 (0.08) [0.00-0.58]	0.04 (0.05) [0.00-0.15]
Younger Black female rate	0.43 (1.02) [0.00-7.50]	1.36 (1.20) [0.00-3.35]
Younger Hispanic female rate	0.10 (0.41) [0.00-4.34]	0.21 (0.49) [0.00-1.42]
Younger White female rate	0.03 (0.07) [0.00-0.35]	0.11 (0.08) [0.00-0.22]

Pregnancy-associated homicide rates of all types were significantly higher where there are documented barriers to finalizing divorce during pregnancy (Table 1). Wilcoxon rank-sum tests indicated this difference is significant for all groups except younger Hispanic females (Table 2).

**Table 2.** Barriers to Finalizing Divorce While Pregnant and Pregnancy-Associated Homicide Rates: Wilcoxon Rank Sum Tests

Variable	Absolute *z* score	*P* value
Intimate partner rate	3.42	.001
Non–intimate partner rate	2.45	.03
Younger Black female rate	3.51	.001
Younger Hispanic female rate	1.66	.17
Younger White female rate	3.87	.001

Spearman correlations reported in Table 3 show more expansive reproductive health care policies were negatively associated with pregnancy-associated homicide rates among younger Black females (ρ = −0.20; *P* = .006) and younger White females (ρ = −0.23; *P* = .002). More-restrictive policies were positively associated with all types of homicide rates, except among younger White females.

**Table 3.** Reproductive Health Care Policies and Pregnancy-Associated Homicide Rates per 100 000 Females of Reproductive Age^a^

Variable	Expansive policies		Restrictive policies		Abortion barriers	
	ρ	*P* value^b^	ρ	*P* value^b^	ρ	*P* value^b^
Intimate partner rate	−0.08	.28	0.22	.003	0.23	.002
Non–intimate partner rate	−0.13	.08	0.22	.003	0.20	.008
Younger Black female rate	−0.20	.006	0.26	<.001	0.28	<.001
Younger Hispanic female rate	0.03	.70	0.22	.004	0.11	.16
Younger White female rate	−0.23	.002	0.03	0.72	0.26	.001

^a^ Mean (SD) [range] number of expansive policies in place across state-years is 9.53 (4.29) [3-20]; mean number of restrictive policies, 0.93 (0.87) [0-3]; and mean number of abortion barriers, 5.38 (2.57) [0-9].

^b^ Spearman correlation used to determine *P* values.

The number of abortion barriers was positively associated with 4 types of pregnancy-associated homicide (except younger Hispanic females). Three barriers were associated with all types of pregnancy-associated homicide except younger Hispanic females: restricted public funding, mandatory waiting periods, and parental involvement (eTable in Supplement 1).

Table 2 and Table 3 suggest support for both hypotheses, but states with documented barriers to finalizing divorce during pregnancy tended to have less reproductive health care access (Figure). To address this correlation, negative binomial regressions were conducted that control for region, include fixed effects for year, and cluster robust SEs by state (Table 4).

**Figure.**
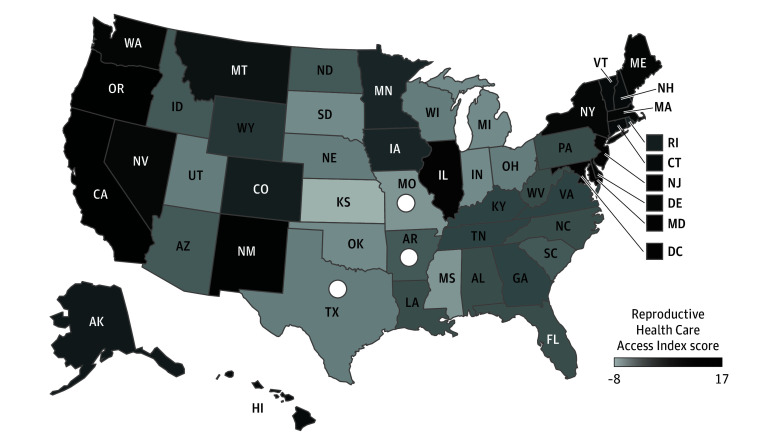
Reproductive Health Care Access White circles identify states with documented barriers to finalizing divorce during pregnancy. The Reproductive Health Care Access Index ranges from −8 to 17; higher scores indicate greater access. For additional information, see the Reproductive Health Care Access subsection of the Methods section.

**Table 4.** Negative Binomial Regression of Pregnancy-Associated Homicide Rates, Barriers to Divorce, and Reproductive Health Care^a^

Variable	IRR (95% CI)	*P* value
**Intimate partner rate**		
Barriers to finalizing divorce	2.11 (1.09-4.08)	.03
Reproductive Health Care Access Index score^b^	0.98 (0.95-1.01)	.19
Southern US	1.49 (0.93-2.39)	.10
**Non–intimate partner rate**		
Barriers to finalizing divorce	0.93 (0.33-2.58)	.88
Reproductive Health Care Access Index score^b^	0.92 (0.87-0.98)	.01
Southern US	1.07 (0.48-2.38)	.87
**Younger Black female rate**		
Barriers to finalizing divorce	2.00 (0.60-6.73)	.26
Reproductive Health Care Access Index score^b^	0.91 (0.87-0.96)	<.001
Southern US	2.17 (1.00-4.71)	.05
**Younger Hispanic female rate**		
Barriers to finalizing divorce	1.07 (0.44-2.58)	.88
Reproductive Health Care Access Index score^b^	0.87 (0.79-0.96)	.007
Southern US	0.87 (0.29-2.58)	.80
**Younger White female rate**		
Barriers to finalizing divorce	2.39 (1.12-5.09)	.02
Reproductive Health Care Access Index score^b^	0.95 (0.90-1.00)	.06
Southern US	3.04 (1.62-5.71)	.001

Abbreviation: IRR, incident risk ratio.

^a^ Total of 181 state-years. Robust SEs are clustered by state; fixed effects for year.

^b^ Information on this index is given in the Reproductive Health Care Access subsection of the Methods section.

Where barriers to finalizing divorce while pregnant were documented, rates of IPH were significantly higher (IRR, 2.11; 95% CI, 1.09-4.08; *P* = .03), as were rates for younger White females (IRR, 2.39; 95% CI, 1.12-5.09; *P* = .02). Rates were not significant for any other racial and ethnic group.

Rates of non-IPH were lower in states with greater reproductive health care access (IRR, 0.92; 95% CI, 0.87-0.98; *P* = .01). Reproductive health care access was negatively associated with homicide rates for younger Black females (IRR, 0.91; 95% CI, 0.87-0.96; *P* < .001) and younger Hispanic females (IRR, 0.87; 95% CI, 0.79-0.96; *P* = .007).

## Discussion

Barriers to divorce while pregnant were associated with higher rates of pregnancy-associated IPH in the US (2018-2021). These results have policy implications for ensuring the safety of pregnant women and highlight the importance of domestic violence resources and safety planning for people leaving abusive marriages. It is crucial that legal support and victim advocates, for instance, ensure pregnant women in abusive marriages secure protective orders and safe housing or other resources, and still *file for divorce,* despite the fact that judges may decide to delay *finalizing* the divorce at that specific stage. As legislators push for changing laws in places like Missouri, there is a clear need for primary research examining the letter and the practice of divorce law in the context of pregnancy in all US states, and on how this is associated with intimate partner homicide.

More pregnant females are killed by an intimate partner than a nonpartner. Training could help health care professionals better identify and respond to signs of partner violence. Pregnant women experiencing abuse should be advised of their heightened risk of pregnancy-related complications and homicide, as well as the effects of violence on their child. Training for gynecologists, obstetricians, and other professionals who administer reproductive services could help them identify reproductive coercion and ensure access to contraceptive methods that are less resistant to sabotage by violent partners. Given that greater reproductive health care access is even more strongly associated with rates of pregnancy-associated homicide perpetrated by someone other than an intimate partner, however, this suggests a need to screen for dangerous contexts other than IPV that place women at risk for pregnancy-associated homicide. This is especially important for groups placed at the highest risk: younger Black and Hispanic girls and women.

Three abortion barriers were associated with higher homicide rates in 4 of the contexts studied, except younger Hispanic females. Rates were higher in state-years with waiting periods, ranging from 18 to 72 hours, suggesting this is a critical time in which medical and criminal justice intervention may be needed. Rates were also higher where parental consent/notification is required, which is of great concern, given it is estimated that approximately 18% of rape-related pregnancies are perpetrated by the female’s father, stepfather, or other family member.^36^ State-years that limit public funding of abortion to life endangerment, rape, or incest also had higher rates, suggesting younger pregnant girls and women who are placed at economic disadvantage are particularly vulnerable due to these restrictions.

While state-level effects were examined, understanding how reproductive health care access varies at the county level and across rural, suburban, and urban divides could reveal economic and geographic factors that facilitate access to care locally and in border states. Understanding reproductive health care resources at local levels could provide resources and points of contact to help ease access, identify signs of violence, and intervene to keep women and girls safe.

### Limitations

This study has limitations. Similar to other datasets on violent deaths, the NVDRS has missing data on key variables, including high levels of missing data on pregnancy status.^8^ Pregnancies among women who experience violent deaths continue to be underreported on death certificates, even with pregnancy check boxes added to these documents in 2003.^37^ Other NVDRS sources may also lack this information depending on the timing of the pregnancy in relation to the woman’s death and whether she had confided in others about the pregnancy. Furthermore, pregnancy-associated homicide rates cannot be calculated for some states due to small numbers, resulting in unstable rates, and data were not available for all states for all years. Given limits to our source material, we cannot determine that there are *no* barriers to finalizing divorce during pregnancy in other states; systematic research in every state is needed on this relationship.

## Conclusions

Laws passed by state legislatures can provide protections or create harms to pregnant women. The findings of this cross-sectional study suggest that homicide rates among pregnant and recently pregnant women vary significantly across state-years in accordance with access to divorce and reproductive health care. Documented barriers to finalizing divorce while pregnant were associated with higher rates of pregnancy-associated IPH, and greater reproductive health care access was associated with lower homicide rates for those killed by someone other than an intimate partner. Reproductive health care access was also associated with lower pregnancy-associated homicide rates among the 2 groups studied at greatest risk: younger Black and Hispanic girls and women.

## Notes

Supplement 1. **eTable.** Specific Barriers to Abortion and Crude Rates of Pregnancy-Associated Homicide per 100 000 Females of Reproductive Age

Supplement 2.
Data Sharing Statement
