# Pervasive Errors Due to Source Material Update

**DOI:** 10.1001/jamanetworkopen.2025.53910

**Published:** 2025-12-23

In the Original Investigation titled, “State Divorce Laws, Reproductive Care Policies, and Pregnancy-Associated Homicide Rates, 2018-2021,”^1^ published November 8, 2024, there were wording errors in the Introduction, Methods, Results, Discussion, Conclusion, and Table 1 and Table 4. A source that was current at original publication was updated 3 months later. The original wording regarding prohibition of finalizing divorce while pregnant has been revised to wording that emphasizes barriers to finalizing divorce while pregnant. The authors have explained what happened in a Comment,^2^ and this article has been corrected.^1^
