# Supplementary material for: State Divorce Laws, Reproductive Care Policies, and Pregnancy-Associated Homicide Rates, 2018-2021
Source: JAMA Netw Open. 2024 Nov 8;7(11):e2444199. doi: 10.1001/jamanetworkopen.2024.44199 (PMC11549657; doi:10.1001/jamanetworkopen.2024.44199)
Supplement: Supplement 1. — eTable. Specific Barriers to Abortion and Crude Rates of Pregnancy-Associated Homicide per 100 000 Females of Reproductive Age [file jamanetwopen-e2444199-s001.pdf]

Supplemental Online Content

Boyle KM, Regoeczi W, Meyer CB. State divorce laws, reproductive care policies, and pregnancy-associated homicide rates, 2018-2021. *JAMA Netw Open.* 2024;7(11):e2444199. doi:10.1001/jamanetworkopen.2024.44199

eTable. Specific Barriers to Abortion and Crude Rates of Pregnancy-Associated Homicide per 100,000 Females of Reproductive Age

This supplemental material has been provided by the authors to give readers additional information about their work.

© 2024 Boyle KM et al. *JAMA Network Open.*

eTable. Specific Barriers to Abortion and Crude Rates of Pregnancy-Associated Homicide per 100,000 Females of Reproductive Age

	Public funding		Waiting periods		Parental involvement	
	|z|	*p*	|z|	*p*	|z|	*p*
Intimate partner rate	2.654	0.007	3.535	< 0.001	2.643	0.007
Non–intimate partner rate	2.003	0.04	3.528	< 0.001	2.458	0.01
Younger Black female rate	3.025	0.002	5.065	< 0.001	4.215	< 0.001
Younger Hispanic female rate	0.989	0.33	1.272	0.20	0.569	0.55
Younger White female rate	2.750	0.004	3.834	< 0.001	2.846	0.003

Notes. N=181 state-years. |*z*| = absolute *z* score. Two-tailed test.

© 2024 Boyle KM et al. *JAMA Network Open.*
