# Supplementary material for: State Divorce Laws, Reproductive Care Policies, and Pregnancy-Associated Homicide Rates, 2018-2021
Source: JAMA Netw Open. 2024 Nov 8;7(11):e2444199. doi: 10.1001/jamanetworkopen.2024.44199 (PMC11549657; doi:10.1001/jamanetworkopen.2024.44199)
Supplement: Supplement 2. — Data Sharing Statement [file jamanetwopen-e2444199-s002.pdf]

Data Sharing Statement

Boyle. State Divorce Laws, Reproductive Care Policies, and Pregnancy-Associated Homicide Rates, 2018-2021. *JAMA Netw Open*. Published November 08, 2024. doi:10.1001/jamanetworkopen.2024.44199

Data
Data available: No

Additional Information
Explanation for why data not available: Data used to create the dataset are publicly available. The coding process by authors will be used in future research and thus will not be made available at this time.
